# DSM-5 Trauma and Stress-Related Disorders: Implications for Screening for Cancer-Related Stress

**DOI:** 10.3389/fpsyt.2013.00122

**Published:** 2013-10-02

**Authors:** Maria Kangas

**Affiliations:** ^1^Department of Psychology, Centre for Emotional Health, Macquarie University, Sydney, NSW, Australia

**Keywords:** cancer, stress, adjustment, anxiety

## Introduction

It is well documented that being diagnosed and treated for cancer is understandably, a challenging experience associated with heightened distress. To this end, in 2009, the International Psycho-Oncology Society endorsed distress as the sixth vital sign in cancer care (1). Indeed, cancer-related distress is common at pivotal periods in the prototypical trajectory of a cancer patients’ experience (including the diagnostic, treatment, recovery, and recurrence phases); and ranges on a continuum from normal, acute responses which may comprise initial fear post-diagnosis, to more severe, potentially chronic stress reactions that adversely impact functionality and general well-being. Therefore, in the Fourth Edition of the Diagnostic and Statistical Manual of Mental Disorders (DSM-IV), being diagnosed with a life-threatening illness such as cancer was included for the first time as a potential traumatic event that could induce posttraumatic stress disorder (PTSD) (2).

Since 1994, there has been a proliferation of studies investigating the prevalence and characteristics of cancer-related PTSD (ca-PTSD) [see reviews, (3, 4)]. The majority of research has been based on self-report questionnaires which has tended to inflate the rates of ca-PTSD (with prevalence rates as high as 55%) compared to studies that have used the gold-standard assessment method of structured, clinical diagnostic interviews (4). In fact, the prevalence rate for ca-PTSD has been documented to be considerably lower when utilizing the latter approach, ranging from 0 to 22% (3). With the Fifth Edition of DSM [DSM-5; (5)], there are some notable changes to screening for stress-related disorders and which have important implications for screening cancer patients and survivors. The purpose of this paper is to evaluate the changes to the PTSD criteria in DSM 5, and the repercussions this has for screening, assessment, and treatment practices for cancer-related stress problems.

## DSM-5: Trauma and Stress-Related Disorders

In DSM-5, disorders which are precipitated by specific stressful and potentially traumatic events are included in a new diagnostic category, “Trauma and Stress-Related Disorders,” which includes both Adjustment Disorders (ADs) and PTSD (5). Friedman and his colleagues (6) assert that there is heuristic value in grouping this set of disorders in a specific stress-related category as it enables clinicians to differentiate between normal (non-pathological) distress, from acute, diffuse clinically elevated stress reactions indicative of AD, to more severe and chronic psychopathology (including PTSD). This heuristic framework also has potential utility for delineating psychological disturbances arising from cancer-related stress. It brings to the forefront the importance of carefully differentiating whether a patient’s stress reactions pertaining to their cancer experience are acute, yet interfering with functioning, indicative of AD, or more severe psychopathology such as PTSD.

Although ADs have been documented to be highly prevalent in cancer patients ranging up to 35% (7, 8), they have tended to be overlooked in studies specifically investigating the prevalence and characteristics of ca-PTSD. Since the publication of DSM-IV, from the studies which have examined the incidence or prevalence of ca-PTSD utilizing clinical diagnostic interviews, only a handful have differentially evaluated the occurrence of partial/sub-threshold PTSD symptoms relative to AD or other anxiety or mood disorders [e.g., Ref. (9–11)]. From this small cohort of studies, the prevalence of ca-PTSD compared to AD has been quite variable. Whereas some studies have found a much higher prevalence of AD relative to ca-PTSD [e.g., 20 vs. 2% (11); 7 vs. 2% (12)], other studies have found ca-PTSD is more prevalent than AD [e.g., 5 vs. 2% (13)]. These mixed outcomes may in part be due to differences in the timing of the assessment (i.e., time elapsed since cancer diagnosis and treatment completion), as well as in diagnostic approaches utilized to screen for ca-PTSD relative to AD and other types of anxiety and mood disorders.

A close inspection of the ca-PTSD literature indicates that a greater proportion of patients meet sub-threshold symptoms for PTSD rather than full diagnostic criteria [e.g., 33 vs. 5% full-PTSD (14); 13.6 vs. 0% full-PTSD (15); 20.3 vs. 16.2% full-PTSD (16)]. In accordance with DSM-IV (2) and DSM-5 criteria (5), an AD diagnosis should be considered for persons who *only* meet partial criteria for PTSD, and if these symptoms are *not* better accounted by another type of anxiety or depressive disorder. However, the majority of cancer studies which have assessed PTSD symptoms, have not considered whether AD, or even another anxiety or mood disorder may better represent the symptom profile for at least some cancer patients who elicit persistent distress for more than one month. To this end, the changes to some core criteria for PTSD in DSM-5 will necessitate a more differential approach to assessing the symptom profile of stress reactions in cancer patients.

## DSM-5 PTSD Criteria: Key Changes and Implications for Cancer Patients and Survivors

Criterion A has been tightened with DSM-5. Importantly, a clear caveat has been included which notes that “*A life threatening illness or debilitating medical condition is not necessarily considered a traumatic event. Medical incidents that qualify as traumatic events involve sudden, catastrophic events*” [(5), p. 274]. Hence, a diagnosis or being treated for cancer *per se* with no adverse events is not necessarily sufficient to qualify for a PTSD diagnosis. Furthermore, for family and friends, “witnessed events” include “unnatural death”; and “learning” about threatening life events must be violent or accidental. Therefore, learning about a relative’s cancer, or death resulting from cancer would not qualify as a PTSD stressor. Similarly, this exclusion criterion is also applicable for persons who learn that they have a genetic vulnerability to developing cancer in terms of carrying a particular cancer gene. In such circumstances, although this information is stressful, if a person elicits heightened, persistent stress reactions, an alternative diagnosis needs to be considered contingent on profile and duration of symptoms, including AD, Generalized Anxiety Disorder, as well considering the relevancy of Illness Anxiety Disorder, or Somatic Symptom Disorder.

If an individual does experience adverse cancer-related events, they would also need to meet the four core criteria that now comprise PTSD (Criteria B to E), instead of the three core clusters stipulated in DSM-IV PTSD criteria. This change is in line with at least 10 published factor analytic studies [see Ref. (17)], including two cancer studies (16, 18), which have found that a four-factor model provides a better fit for the PTSD construct. This has the propensity to tighten the sensitivity threshold in identifying persons whose stress reactions reflect PTSD relative to other anxiety and mood disorders. In particular, the four core PTSD clusters require that an individual elicits at least one of five re-experiencing symptoms (Criterion B), one of two avoidance symptoms (Criterion C), two of seven dissociative and/or negative cognitive symptoms (Criterion D), and two of six arousal and reactivity symptoms (Criterion E).

Although minimal changes have been proposed for the re-experiencing (Criterion B) cluster, the reporting of intrusive symptoms need to be evaluated in relation to “*involuntary and intrusive distressing memories*” (Criterion B1). This is in contrast to individuals worrying about their future health including fear of cancer recurrence. If these latter responses better defines a person’s intrusive reactions then these symptoms do not qualify for ca-PTSD. Similarly, Criteria B2, B4, and B5 (distressing dreams, emotional, and physiological) symptoms need to also be assessed in context of AD and other anxiety disorders, particularly if the individuals fears are primarily future-oriented.

In DSM-5, separating the PTSD avoidance from the numbing and cognitive symptoms into two distinct clusters (Criteria C and D respectively) has the potential to further reduce the rates of false-positive ca-PTSD. Previously, according to PTSD-DSM-IV Criterion C, a person with cancer could have met this criterion without reporting avoidance reactions. However, individuals must now elicit at least one avoidance symptom to meet Criterion C and which is directly related to avoidance of “distressing cancer memories” (i.e., cancer-events that have transpired).

The proposed new Criterion D (numbing/dissociative and negative cognitive reactions) also has the scope to enhance diagnostic sensitivity and specificity. Three of the seven symptoms (Criterion D2, D3, and D7) are new or amended criteria. Criterion D2 has been modified from “a sense of foreshortened future” (Criterion C7, PTSD-DSM-IV) and expanded to capture negative distorted attributions about the self, others, or the world post-trauma. Criterion D3 assesses whether a person elicits unrealistic blame of self or others pertaining to the cause or consequences of their stressor experience. However, attributions of self-blame may be grounded in reality for some cancer patients. For example, a person who has been a chronic smoker and subsequently develops lung cancer may understandably elicit self remorse for their lifestyle choice. However, if these symptoms are accompanied by exaggerated self-deprecating schemas, this may also be indicative of depression. Criterion D4 expands upon the previous subjective Criterion A2 (in DSM-IV) in order to capture a wider range of pervasive emotional reactions post-trauma which includes feelings of guilt, shame, and anger. The four remaining Criterion D numbing and dissociative symptoms (D1, D5, D6, and D7) remain the same from DSM-IV, PTSD criteria.

The fourth cluster, Criterion E now includes reactive as well as arousal symptoms. Specifically, Criterion E1 has been expanded to include unprovoked anger outbursts, and the new Criterion E2 captures reckless or self-destructive behavior. Although these symptoms have been reported in veteran and non-medical trauma samples, there is a paucity of studies that have indicated whether such symptoms also arise in distressed cancer patients.

Furthermore, although the criteria for duration (Criterion F) and functionality (Criterion G) have remained the same in DSM-5 (i.e., the constellation of symptoms endure for more than 1-month, and result in impairment in one or more areas of interpersonal and/or occupational functioning), the expansion of the core criteria to four core clusters, with particular emphasize of involuntary and distressing intrusive memories is likely to reduce the prevalence of ca-PTSD. This is because there is an increasing body of literature that demonstrates that most distressed cancer patients tend to be worried about current and future health concerns, including fears of cancer recurrence [e.g., Ref. (19)], instead of eliciting and avoiding intrusive, involuntary, distressing cancer-related memories. Moreover, with the inclusion of AD in the new category of Trauma and Stress-Related Disorders, it is timely for the psycho-oncology field to re-consider the utility of AD as well as other anxiety and mood disorders in screening for clinical stress reactions in cancer patients. Indeed, the DSM-5 stipulates that ADs “*are common accompaniments of medical illness and may be the major psychological response to a medical disorder*” [(5), p. 289].

## Conclusion

With the introduction of the “Trauma and Stress-Related Disorders” category in DSM-5, the pertinent issue is *not* whether AD better defines clinically elevated cancer-related stress reactions compared to PTSD, or whether either or both of these frameworks have utility for defining heightened ca-related distress beyond other mental health disorders. Rather, these changes have important clinical implications in identifying cancer patients and longer-term survivors who are experiencing psychopathology. It is recommended that clinicians (and researchers) carefully consider differential diagnostic parameters as well as screening for psychological history (pre-cancer diagnosis) when evaluating stress reactions in cancer patients. Indeed, most psycho-oncology studies to date have overlooked screening for patients’ psychiatric history, resulting in probable overestimation of prevalence rates. To this end, screening patients’ psychiatric history is important to ascertain whether distress reactions elicited post-cancer diagnosis are an exacerbation or recurrence of pre-existing mental health problems (including other anxiety and depressive disorders) compounded by the stress of adjusting to one’s cancer diagnosis. Moreover, this has important treatment implications in ensuring an appropriate evidence based therapy is recommended to patients. For example, presently, the strongest supported evidence for the treatment of PTSD is Trauma-Focused Cognitive Behavioral Therapy (TF-CBT) which includes imaginal exposure targeting sub-traumatic memory pertaining to the trauma experience (20). If however, a cancer patient’s diffuse stress symptoms are misdiagnosed as ca-PTSD, applying an imaginal exposure component as part of a TF-CBT program may be ineffective and lead to poor or delayed treatment gains. Hence, accuracy in diagnosing ca-PTSD relative to AD and other types of anxiety and mood disorders has direct implications for selecting appropriate treatments for client benefits. It is therefore timely with the shift to DSM-5, that a more comprehensive approach in screening for debilitating stress problems in cancer patients is utilized, which takes into account patients’ psychological history.
